# An Internal Medicine Learning Collaborative Facilitating a Virtual Continuing Medical Education Program in Guyana and the Wider Caribbean During the COVID-19 Pandemic

**DOI:** 10.7759/cureus.56972

**Published:** 2024-03-26

**Authors:** Balichand Permashwar, Jagindra Mangru, Eric Yu, Katherine M Spencer, Karen Goble, Mayank Singhal

**Affiliations:** 1 Hospital Medicine, Cape Fear Valley Medical Center, Fayetteville, USA; 2 Hospital Medicine, FirstHealth of the Carolinas, Pinehurst, USA; 3 Medicine, Campbell University School of Osteopathic Medicine, Lillington, USA; 4 Internal Medicine, Cummings Rheumatology, Atlanta, USA; 5 Internal medicine, Campbell University School of Osteopathic Medicine, Fayetteville, USA; 6 Neurology, Cape Fear Valley Medial Center, Fayetteville, USA; 7 Medicine, Southern Regional Area Health Education Center (SR-AHEC), Fayetteville, USA; 8 Internal Medicine, Cape Fear Valley Medical Center, Fayetteville, USA

**Keywords:** free, online, covid-19, caribbean, guyana, cme, collaboration

## Abstract

Objectives: To collaborate and share medical knowledge between US and Caribbean physicians during the COVID-19 pandemic via a free online continuing medical education (CME) series.

Method: This was a multi-institution collaborative effort between the Southern Regional Area Health Education Center and Cape Fear Valley Medical Center, both located in North Carolina, USA, and its Caribbean partners, the Guyana Medical Council and Ministry of Health, and the University of the West Indies Medical Alumni Association, Jamaica. The lecture series ran from July 2021 to October 2022. The Zoom (Zoom Video Communications Inc., San Jose, CA, USA) meeting platform was used for the monthly lectures on the fourth Thursday between 7 and 8 p.m. Eastern Standard Time (EST).

Results: Analysis of program data from July 2021 through October 2022 (excluding December 2021) found 1,105 unique individuals engaged in the 15 continuing education sessions. The series had a cumulative total of 2,411 participants, with a mean session participation of 161 and a range of 94 to 299 participants per lecture. An outcome survey assessing the reasons for attendance identified that the most significant factors in their participation in the series were: a) the quality of educational content (83.21%), b) the ease of access and Zoom platform (81.76%), and c) the lectures being offered at no cost (61.31%), and 80.84% gained new medical knowledge leading to practice changes.

Conclusion: The Internal Medicine Learning Collaborative (IMLC) model can be easily replicated by following the steps outlined. It overcomes barriers such as travel and quarantine restrictions and is cost-effective to initiate and maintain. It allows physicians with access to resources and specialty training in the United States to share medical knowledge with colleagues in the developing world where such access may be limited, thus promoting health care and continuing education activity in their respective regions using freely available technologies.

## Introduction

It is well known that the COVID-19 pandemic stressed an already strained healthcare infrastructure worldwide [1,2]. The term 'healthcare infrastructure' encompasses the various resources that deliver healthcare services to meet the health needs of the target population, including hospitals and institutions responsible for training residents and student doctors through graduate and undergraduate medical education. In the United States, COVID-19 influenced the transition of pre-clinical medical students to online medical education, while those on clinical rotations had many of these rotations canceled. Graduate medical education transitioned to digital platforms for didactic sessions and many resident physicians were redeployed from non-critical care specialties to the front of the pandemic [3-5]. The COVID-19 pandemic disproportionately affected minority communities in both the United States and in low- and middle-income countries (LMICs) worldwide [6]. Subsequently, a dire need for continuing medical education (CME) to stay up-to-date on the treatment modalities for COVID-19 was identified in those regions. During the initial phase of the COVID-19 pandemic, high-efficacy vaccines (>90%) such as Moderna and Pfizer/BioNTech were not readily available to LMICs. This lack of availability for these countries has been tied to the global governance of pharmaceutical ingredients and manufacturing capabilities [7].

Herein, we review our experience with establishing a collaborative CME series engaging providers in an underserved region of North Carolina and LMICs. We also discuss the sustainability of such programs and hope to create a reproducible model by sharing our reflections on this experience and the current literature.

## Materials and methods

Identifying gaps in education and healthcare 

In early 2021, colleagues working in Guyana shared their difficulties treating critically ill patients given the lack of resources, such as critical care, infectious disease, and pulmonology, and the lack of readily available specialist physicians to consult. The few local specialist physicians available were facing the same logistical challenges. Guyanese physicians had access to treatment protocols and some specialist help from organizations such as the Pan American Health Organization (PAHO) and the WHO. However, they needed more support at ground level. There is no direct comparison between our practice in North Carolina and those of LMICs; however, we do have some shared experience as we practice in a region of the state with the poorest health outcomes and highest risks regarding social determinants of health [8].

In March 2021, a medical symposium was organized between US physicians working at Cape Fear Valley Health Systems (CFVHS), North Carolina, and the infectious disease hospital in Guyana to share our experiences in managing COVID-19 infections. The meeting was attended by a hospitalist physician, an infectious disease physician, an intensive care physician, and our Guyanese colleagues via Zoom (Zoom Video Communications Inc., San Jose, CA, USA), an accessible video-conferencing platform. The topics discussed were broad but included the pathophysiology of the COVID-19 infection, appropriate serum biomarkers, radiologic tests to order, medication options, different oxygen delivery modes, and mechanical ventilation modes. The talk was considered a success, with over 120 participants involved, and was extended to accommodate numerous questions and share treatment approaches by both groups. At the time of the symposium, we found our Guyanese colleagues were up to date with the current medical literature about the treatment of COVID-19 infection but often lacked access to the required laboratory, radiologic services, and equipment, such as mechanical ventilators, which at that time was a worldwide problem [9]. The newly built infectious disease hospital in Georgetown, Guyana, was still being equipped, and specialized radiologic tests such as CT and computed axial tomography (CAT) scans were done off-site, which was difficult to achieve with critically ill patients. Guyanese physicians valued this exchange and expressed an interest in further collaboration through an online general medicine CME program since in-person activities were suspended. Coincidentally, CFVHS providers also experienced suspension of their quarterly Internal Medicine Grand Rounds due to the pandemic and also needed clinical updates, which led to the creation of the Internal Medicine Learning Collaborative (IMLC) program.

Building relationships locally and in the host region

We approached the Ministry of Health, Guyana, and the Guyana Medical Council (GMC) with the idea of starting a collaborative, regularly scheduled series, and both were in agreement. During the height of the pandemic, we had to overcome a lack of experience regarding the planning of the lecture series, accreditation, hosting, the technology platform to use, and finding speakers. These barriers were overcome by collaborating with the Southern Regional Area Health Education Center (SR-AHEC) in North Carolina and their teaching hospital partner, Cape Fear Valley Medical Center (CFVMC), also in North Carolina. An Institutional Review Board (IRB) application was sent to CFVMC under the non-human research category, and our lecture series was approved (approval no. 1182-24). Hence, the monthly CME series, the IMLC, was born. The first and last authors were the co-medical directors, and the first, second, and fifth authors were responsible for creating the lecture series. 

The initial lectures were conducted by the founders of the IMLC, and subsequently, additional speakers were recruited through word-of-mouth among medical professionals in North Carolina, the Caribbean physician network, and volunteers who knew of the series. All presenters were board-certified in their respective fields and are currently practicing clinical medicine, volunteering their time and effort free of charge. Speakers came from diverse clinical experiences, including attending physicians, a department chair, two fellows, and two Rhodes scholars.

Collaborating with local institutions for accreditation

The SR-AHEC accredited the series and awarded the American Medical Association (AMA) Physician's Recognition Award (PRA) category (Cat) 1 credit for US attendees and contact hours for Caribbean participants. The GMC was able to provide reciprocity of their own accredited CME hours for Guyanese physicians. The GMC received a copy of the lecture two weeks before the presentation date for approval and reviewed the CVs of the speakers and their teaching objectives. All lectures were screened for medical relevance, bias, and conflict of interest by the SR-AHEC CME department and simultaneously by the GMC. Guyanese physicians are required to obtain 12 Cat 1 CME hours annually to maintain their medical licenses, and attending our monthly lectures would fulfill this requirement. Speakers affiliated with the Caribbean were a priority to keep the lecture series relevant to the region. Local literature was incorporated when available, and discussions regarding treatment guidelines were focused on the region's limited resources (CT scans and laboratory tests such as D-dimer, ferritin, and procalcitonin tests are not widely available). With the success of the IMLC in Guyana, the authors were approached by physicians to offer the lecture series to the broader Caribbean. We approached the University of the West Indies Medical Alumni Association (UWIMAA), Jamaica, with the proposal of expanding our lecture series to the entire Caribbean. The University of the West Indies is the premier healthcare organization in the Caribbean, responsible for the accreditation of many local territories, and has numerous distinguished graduates practicing worldwide. The UWIMAA joined the IMLC collaborative and utilized their 'Skill and Soft Life' lecture series for alumni to showcase their work. The series was thus expanded to include all English-speaking Caribbean physicians.

Engineering the delivery of the lecture series

The lecture series ran from July 2021 to October 2022. The series was hosted on the Zoom platform from 7 to 8 p.m. Eastern Standard Time (EST) on the last Thursday of the month for consistency. The CME department of SR-AHEC hosted all lectures, opening the Zoom meeting room 30 minutes before to allow participants to log in and presenters to do an audio and slide check. All lectures had PowerPoint (Microsoft Corp., Redmond, WA, USA) presentations, and the slides were available to the audience upon request. An attendance record was kept, and this was shared with the GMC for Guyanese physicians. The medical director's and SR-AHEC CME director's emails were available on the digital flyer for any questions and clarifications afterward. Lectures were typically 50 minutes long, with 10 minutes for questions and answers. We used Zoom because it was free for participants, widely used in the Caribbean, and did not require additional software to access the lecture series. The SR-AHEC created and distributed a digital flier for US participants and sent a copy to the GMC, who emailed the announcement to registered Guyana physicians, and to the UWIMAA, who emailed it to registered medical alumni worldwide. We realized that the technology we take for granted, such as consistent electricity supply and high-speed broadband internet, was not readily available in LMICs. Also, large countries and regions, such as the Caribbean, have different time zones that had to be specified on the invitation flyer.

Reassessing goals

As the pandemic weakened, other academic medicine programs began hosting CME series online and on-site in the Caribbean during the program's second year. As local participants identified competing priorities, CFVHS and North Carolina provider participation declined during the second year. The decrease in North Carolina physician participation and the new resources for CME in the Caribbean led to a re-assessment of the need and viability by the planners. The need did remain for a virtual CME series for international and Caribbean physicians; however, with dwindling US physician attendance, we could not establish a need and meet the criteria to provide AMA PRA Cat 1 credit for US physicians. This accreditation was a strong reason we were able to attract highly qualified speakers to the program.

## Results

**Figure 1:**
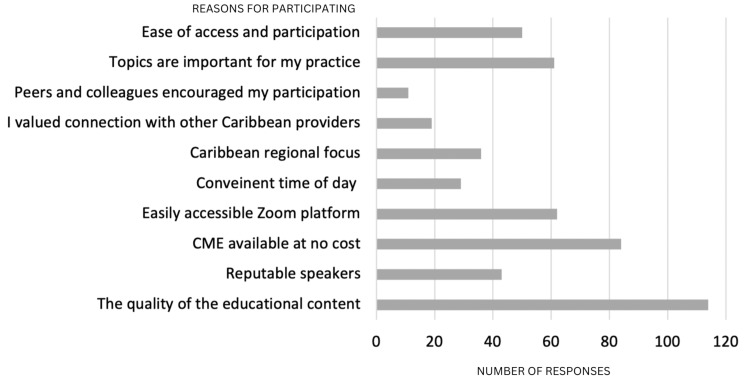
Participants choose the top three reasons for participating in the IMLC IMLC: Internal Medicine Learning Collaborative

**Figure 2:**
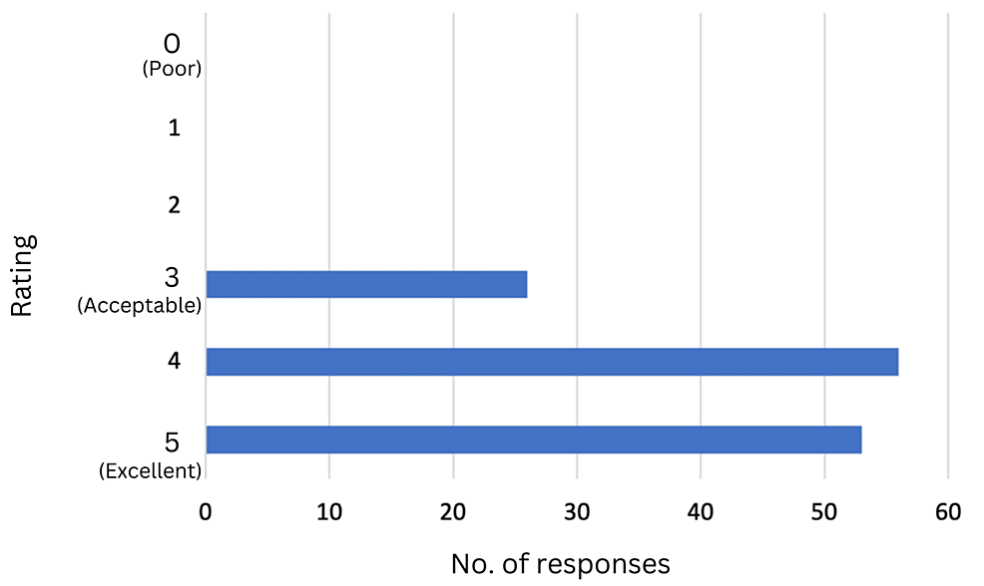
Participants rank the series on a scale of 1 to 5, indicating how well the series updated their knowledge of clinical practices.

Analysis of program data from July 2021 through October 2022 (excluding December 2021) found 1,105 unique individuals engaged in the 15 IMLC CME sessions and benefiting from the activities. The series had a cumulative total of 2,411 participants, and mean session participation was 161, with a range of 94 to 299 participants (Table 1). Four of the 15 sessions had over 200 participants. Most participants (720, 65.1%) engaged in two or more sessions supporting the educational format as a regularly scheduled series. Further analysis examining US providers found that 36 had been awarded credit in the first year and only 11 in the second year (additional North Carolina healthcare providers participated and were identified on the Zoom roster but did not seek credit). The CFVHS provider participation was consistent with the prior internal medicine series. In June 2022, the SR-AHEC CME distributed an outcome survey via email to the 941 series participants that, as of May 2022, yielded 137 responses (14.56%). Respondents identified that the most significant factors in their participation in the series were: a) the quality of educational content (83.21%), b) the ease of access and Zoom platform (81.76%), and c) the lectures being offered at no cost (61.31%) (Figure 1). Most respondents worked in hospitals or clinics. Others were involved in education, public health, or rehabilitation; one reported serving on an offshore rig. Notably, 80.84% of respondents confirmed that they had made a change in practice during the year based on CME (Figure 2). The general feedback from participants was that the series successfully met their educational needs. The presenters also found this to be a highly rewarding experience; the sharing of information and preserving human contact at a time of isolation were of mutual benefit.

**Table 1:** The participant breakdown of our IMLC sessions IMLC: Internal Medicine Learning Collaborative

Particulars	Value
Number of total unique participants	1,105
Cumulative total number of participants	2,411
Mean session participation	161
Range of participants per lecture	94 to 299
Number of sessions	15

## Discussion

Reflections on the current literature

Guyana is a small English-speaking country considered to be an LMIC on the Atlantic coast off of South America, with a burgeoning oil and gas economy and a population of approximately 750,000 [10]. Although the healthcare sector in Guyana is growing, several gaps remain, including the lack of access to specialty services and CME for physicians, especially during the early pandemic. To overcome these barriers, hospitals in Guyana are collaborating with international universities to develop local teaching programs, share knowledge, and gain international experience. The need for further graduate medical education in Guyana became increasingly evident during the COVID-19 pandemic [11].

The current literature reflects a need for increased availability of CME in LMICs like Guyana. Guyana has 2.1 physicians for every 10,000 people. In perspective, Canada has 2.1 physicians for every 1,000 people, and the US has approximately three physicians for every 1,000 people [12]. In 2017, Persaud et al. and Stokes et al. described creating the internal medicine/infectious disease residency program in Guyana in collaboration with the Georgetown Public Hospital Corporation (GPHC) to address healthcare worker shortages and the 'brain drain' phenomenon, i.e., over 75% of individuals with post-secondary education leave Guyana for other countries. In addition, medical students and residents often pursue specialist training outside the country, rarely returning to Guyana [11,13]. This problem is not unique to Guyana. Other LMICs struggle to educate their healthcare workers and medical students due to a lack of organized medical education. In 2018, Wondimagegn et al. described a continuing, successful partnership between the University of Toronto in Canada and Addis Ababa University in Ethiopia, emphasizing the steps taken to form the partnership [12]. Wondimagegn et al. partnered to mitigate the 'brain drain' from Ethiopia. Similar motifs have been seen in long-term collaborations between high-income countries and LMICs like Laos, Uganda, and Kenya [14-16]. A Best Evidence Medical Education (BEME) systematic review of continuing professional education in LMICs was published in 2021, describing the key to developing successful collaborations and the common pitfalls of such programs. Hill et al. described common barriers in their systematic review, including Western bias and cultural differences. Five progressive stages were identified that collaborators might need to navigate to have a successful and equal collaboration: personal values, relationships, resilience, benefits, and feed-forward [17]. We found that the systematic review by Hill et al. provides an excellent framework for creating a collaborative program and can provide insight into current and past collaborations.

Reflections on the IMLC program

While the literature describes the collaboration between high-income countries (HICs) and LMICs, most of these collaborations refer to grant-funded programs in which providers from the HIC travel to assist with teaching health professionals. Our IMLC was created to expand the CFVMC internal medicine CME, in light of the COVID-19 pandemic, to providers in Guyana, which eventually expanded to the Caribbean. As such, our collaboration did not fall prey to certain financial drawbacks described by Hill et al., namely the inability to approve project funding. However, our IMLC did have its challenges and pitfalls, which we describe in Table 2.

**Table 2:** Barriers encountered during the IMLC program and the solutions implemented Note: Not all barriers had immediate solutions. PAHO: Pan American Health Organization

Barriers	Solutions
Initially, there was a lack of experience regarding the formation of the lecture series, accreditation, hosting, the technology platform to use, and finding speakers.	Via networking (relationships) with the hospital's CME department, and university and medical school alumni, we found speakers and obtained AMA PRA Cat 1™ accreditation for US providers.
It was a challenge to organize programs and collaborate with overseas partners during a pandemic while performing regular medical duties on both sides.	The mutual benefit of collaborating during the pandemic helped overcome isolation, and preserving person-to-person contact was highly rewarding hence our perseverance.
Inequities between high-income countries and LMIC treatment modalities, e.g., the lack of many diagnostic tests and treatment modalities for the treatment of diseases made it difficult to provide practical medical therapies.	Providers offer treatment modalities tailored to the region.
Gaps with Caribbean partners disseminating meeting details promptly affected attendance at some of the sessions.	Past participants who provided their email addresses were directly emailed flyers advertising the time and topic for that day.
Insufficient population health and disease-specific data, including available treatment options in the Caribbean and Guyana, made it challenging to address; shared practice while noting local conditions and limitations.	General population data was available at PAHO and WHO websites and local studies in the West Indies Medical Journal were incorporated when appropriate.
We could not meet all specific topics requested by audience feedback due to dependence on speakers volunteering their time for free while still performing their regular duties.	We were able to meet some requests relevant to adult medicine. We focused on our speaker’s specialty and chronic diseases where local specialist help was minimal.

Our IMLC ultimately concluded towards the end of the COVID-19 pandemic due to the changing needs and priorities of US attendees and not due to the lack of foreign participants. The AMA PRA Cat 1 accreditation was important in recruiting quality speakers for our series. When our lecture series ended, there was still an identified need for CME for international and Caribbean physicians. While international and Caribbean physicians have their own accreditation process depending on their home country, physicians holding American Board Certification need CME accreditation by the Accreditation Council for Continuing Medical Education (ACCME). Unfortunately, the response rate for the surveys sent out to participants was low, at 14.56%. This was most likely due to a lack of incentive to complete the survey after participating in the lecture and the pressures experienced by providers in all countries during the pandemic. The survey was done approximately one year after the series began, and no barriers were placed to obtaining credit as our goal was to share knowledge and not collect data. The lectures were provided at no cost to participants, and the collaboration ensured no barriers were present to obtaining CME or contact hours from attending the talks.

## Conclusions

Overall, the IMLC series met the intended goals of collaboration and sharing of medical knowledge, which led to practice changes and an international exchange during the pandemic isolation. We believed it was successful because of the collaborative bridges we built, the sharing of knowledge and experience, and the high attendance rate. We did face limitations as this series was conducted during the chaos of the COVID-19 pandemic; creating the series and finding speakers and institutions to volunteer their services free of charge was at times challenging. The series can be replicated by following the steps outlined and will be easier to implement in the non-pandemic era. We recommend implementing the survey earlier and analyzing the collected data to help with self-reflection and guidance.

We share this experience with the hope of inspiring other much-needed collaborations to help address the gaps in our healthcare and CME provision in countries with limited resources. Improving infrastructure, such as hospitals and equipment, in underdeveloped countries is often financially challenging. However, access to knowledgeable, well-trained, and resourceful healthcare providers can offset the need for more material infrastructure to deliver good healthcare. The virtual CME series is a cost-effective means of strengthening the human resource component of the healthcare system. We can use the ubiquitous social media and internet to promote medical education beyond borders. The IMLC is also an excellent opportunity for specialists to share their knowledge and expertise with a broader population from the comfort of their homes. It can be easily replicated and is a meaningful way to give back to the community and help improve patient care.
